# Motor-Cars for Doctors

**Published:** 1906-05-26

**Authors:** 


### HOSPITAL ADMINISTRATION.

### CONSTRUCTION AND ECONOMICS.

### MOTOR CARS FOR DOCTORS.

GENERAL DESCRIPTION.

BEFORE closing the subject, it may perhaps be of service to members of the medical profession if a description is given of the general arrangement of all motor-cars. Full details of the different forms will be found at great length in the books devoted to the subject. As indicated in a previous article, the power for driving motor-cars is obtained either from petrol engines, steam engines, or electric motors. In petrol engines the power is obtained from petrol by the explosion of mixtures of petrol vapour and air within one, two, or more cylinders. With steam the power is obtained from steam generated in a boiler, or steam generater, as the makers prefer to call it, by the action of petrol vapour and air burning, as in an ordinary boiler furnace. In both cases the power is transmitted to a revolving axle, called the crank shaft, and from it to the rear wheels of the car by various devices. With the electric car the power is obtained from a battery of accumulators carried on the car, which have to be charged from a dynamo machine after every journey, and which should always be charged after standing without running for a few days.

There is a considerable difference between the methods of transmitting the power from the crank shaft to the wheels in the petrol and steam cars. With steam cars the engine can be made to run in either direction, and the pressure at which the steam enters the cylinder can be regulated at will; and so it follows that the crank shaft is connected directly to the gear wheels which come immediately in front of the driving wheels—the differential gear, as it is called, the arrangement provided to enable the car to go round sharp curves, by allowing the two wheels, which will then be going at different speeds, to run independently of each other. With the petrol car, however, the engine only runs in one direction, and arrangement has to be made to disconnect the crank shaft from the remainder of the gear when the car is standing or when it is required to reverse. The system of wheels, clutches, etc., is known generically as gear. The disconnection is accomplished by a clutch (an apparatus well known in mechanics), in which two surfaces, connected to the two parts that are to be made to run together, are brought into close connection or pulled apart, the clutch being put in operation by a powerful spring and held back by a pedal on the driver's seat. The petrol engine, also, will not start by itself as the steam engine will, so a handle is provided, a few turns of which cause the crank shaft to revolve and the explosions to commence. In addition, it is necessary with the petrol engine to provide for varying the speed at which the car wheels are revolving, apart from the speed at which the engine is running. This is accomplished (in some cars) by a system of wheels contained in an iron box and running in oil. The speed of the engine is reduced, in its transmission to the wheels, in different proportions, by different sets of toothed wheels meshing together, and the gear wheels in the box are arranged to assume different meshings in obedience to the operation of levers at the driver's hand. There are four separate axles in the gear box, each carrying toothed wheels that can be brought into mesh with other toothed wheels at will, one set giving the first speed, another the second, while the elimination of all the toothed wheels gives the highest speed, that at which the engine itself is running. Another little toothed wheel that is idle, except when performing its particular work, gives the reverse motion when required for running the car backwards. It is merely a wheel interposed between those that are transmitting the power. Some cars vary the speed by means of what is called epicyclic gear, which is claimed to be more efficient —that is, to deliver a larger proportion of the power of the engine at the car wheels. It consists of a system of wheels revolving inside a larger wheel having teeth on its inner surface. There is a centre wheel and three or four wheels connecting the centre wheel with the outer wheel, and it is arranged by different meshings to obtain different speeds. The cars employing epicyclic gear have their engines usually horizontal under the car; those using spur wheels, where several are enclosed in a box, usually have the engine, which is vertical, under the bonnet in front. Sprocket chains and wheels are also employed in transmitting the power in some cars. They consist of wheels having long projecting teeth which fit into the links of a chain which passes over them, carrying the power from one wheel to another and reducing the speed in the process. In addition to the above, the petrol car carries a tank of petrol generally sufficient for 100 to 150 miles run; an electrical apparatus for providing the spark that ignites the charge of petrol and air and causes the explosions, and for arranging that it passes at exactly the right time; and, in modern cars, an arrangement for advancing or retarding the time. Also what is called a carburettor is required—the apparatus which converts the petrol into vapour, either by heat or by breaking it up into a fine spray. It is only petrol vapour that will mix with air, and will either explode or burn, to raise steam. A cooling apparatus is also carried for keeping the engine cylinders and valves within working temperatures. It consists of a tank of water connected with linings on the outsides of all the engine cylinders and with what is called a radiator (the apparatus seen in the front of each petrol car), consisting of tubes through which the water is either pumped, or passes by gravity, the outsides of the tubes being exposed to the current of air made by the car in travelling, assisted in some cars by a small fan immediately behind the radiator, driven by the engine. The cooling effect of the radiator enables a much smaller quantity of water to be carried, as the water is used over and over again. The steam car carries an apparatus similar to the radiator of the petrol car. It is called the condenser, and its office is to convert the exhaust steam which has done its work in the engine into water, which is pumped back to the water tank and used over again in the boiler. The petrol car requires yet one more apparatus, the silencer. This is a cylinder, carried horizontally under the car, into which the exhaust gases from the engines are turned, and in which the unpleasant coughing noise is extinguished. It simply uses up the energy remaining in the gases, in place of allowing them to expand into the atmosphere. Steering is performed in all motor cars by turning the front wheels on a pivot, the turning being usually accomplished by means of the familiar wheel which the driver holds. To the steering wheel are frequently attached levers for controlling different parts of the apparatus, while other levers are fixed within easy reach of hand or foot, so that the driver has complete control—far more perfectly than with the horse—of every motion of the car from his seat. The tendency in modern petrol cars is more and more to run on the top speed gear without bringing the toothed gear into use, controlling the actual speed by the quantity of petrol vapour and air that is admitted to the engine at each stroke. This saves the gearing very much, and is much better and more efficient; but the gear has to be used in all cases with petrol cars when it is required to run backwards. Steam or electric cars can run backwards or forwards at will by altering the direction of flow of the current or the steam. Electric cars are controlled by an apparatus at the driver's hand, somewhat similar to the controller of an electric tramcar, the controller altering the grouping of the accumulator cells and the direction of the current passing into the motors.

